# Paced-Mating Increases the Number of Adult New Born Cells in the Internal Cellular (Granular) Layer of the Accessory Olfactory Bulb

**DOI:** 10.1371/journal.pone.0019380

**Published:** 2011-05-27

**Authors:** Rebeca Corona, Jorge Larriva-Sahd, Raúl G. Paredes

**Affiliations:** Instituto de Neurobiología, Universidad Nacional Autónoma de México, Querétaro, México; Duke University, United States of America

## Abstract

The continuous production and addition of new neurons during life in the olfactory bulb is well accepted and has been extensively studied in rodents. This process could allow the animals to adapt to a changing environment. Olfactory neurogenesis begins in the subventricular zone where stem cells proliferate and give rise to young undifferentiated neuroblasts that migrate along the rostral migratory stream to the olfactory bulb (OB). Olfaction is crucial for the expression of sexual behavior in rodents. In female rats, the ability to control the rate of sexual interactions (pacing) has important physiological and behavioral consequences. In the present experiment we evaluated if pacing behavior modifies the rate of new cells that reach the main and accessory olfactory bulb. The BrdU marker was injected before and after different behavioral tests which included: females placed in a mating cage (control), females allowed to pace the sexual interaction, females that mated but were not able to control the rate of the sexual interaction and females exposed to a sexually active male. Subjects were sacrificed fifteen days after the behavioral test. We observed a significant increase in the density of BrdU positive cells in the internal cellular layer of the accessory olfactory bulb when females paced the sexual interaction in comparison to the other 3 groups. No differences in the cell density in the main olfactory bulb were found. These results suggest that pacing behavior promotes an increase in density of the new cells in the accessory olfactory bulb.

## Introduction

The continuous addition of new cells during life has been found in several brain regions including: different cortical areas, the olfactory tubercles, anterior olfactory nuclei, tenia tecta, islands of Calleja, amygdala, caudate nucleus and nucleus accumbens [1], [2], [3], [4]. However, the most studied regions that incorporate new neurons in adulthood are the hippocampus and the olfactory bulb (OB) [5], [6]. In the hippocampus these new cells are born in the dentate gyrus and integrate into the granular layer. In the olfactory bulb this process has been extensively described in mice and consists of three stages: proliferation, migration and survival of the new cells. Proliferation begins in the subventricular zone (SVZ) located in the walls of the lateral ventricles, where most of the cells are generated. The process begins with the astrocyte-like stem B cells that give rise directly to neuroblast A cells that migrate tangentially along the rostral migratory stream (RMS), where a few cells can also be generated [7], [8] to finally reach the OB [9], [10]. Once in the OB they migrate radially to the granular and glomerular layers of the structure and integrate as mature interneurons [11], [12], [13], [14].

The new cells that proliferate in the SVZ reach the main (MOB) and the accessory olfactory bulb (AOB) 15–20 days later [15], [16], [17], [18]. It has been suggested that the OB continuously needs to renew its cells as an adaptive mechanism to allow the animals to discriminate different types of odors in a continuously changing environment. For example, it has been described that odor stimuli can modify the rate and survival of adult neurogenesis in the OB [15], [19]. Exposure to males increases the number of BrdU-labeled cells in the SVZ of the female prairie vole [20]. It has also been shown that the new neurons that migrate and incorporate into the OB after mating or after exposure to female vaginal secretions in golden hamsters are Fos immunoreactive (as a marker of neuronal activation) [21] indicating that these new cells that reach the OB are functional. Mak et al. [22] reported that neurogenesis in the OB is correlated with the preference of female mice for dominant males, since pheromones of the preferred dominant male stimulate cellular proliferation in the SVZ and neuronal production in the MOB. These results suggest that neurogenesis in the OB may play a significant role in detecting sociosexual cues and in mating behavior, but the actual role of these new OB cells in social behaviors is still unknown [15], [23].

The ability of female rats to control the rate of sexual interactions (pacing) has important physiological [24], [25], [26] and behavioral consequences [27], [28]. Pacing provides a behavioral mechanism by which females maximize the genitosensory stimulation needed to induce neuroendocrine changes [24], [25], [26]. For example, fewer intromissions after paced than after non-paced mating are required to induce physiological changes [27]. We have also shown that when females paced their sexual interaction a reward state evaluated by conditioned place preference (CPP) is observed [29], [30]. This reward state is mediated by opioids since intracerebral infusion of the opioid antagonist naloxone into regions involved in odor processing, such as the medial preoptic area (mPOA) and the amygdala (Me), blocks the reward state induced by paced mating [31]. Moreover, whenever given the opportunity, females will control the rate of sexual interaction in laboratory settings [28] and in seminatural conditions [32], [33] further supporting the importance of pacing the sexual interaction for female sexual behavior.

The present study examined whether pacing behavior could somehow influence the density of new cells that arrive to the OB. Using the mitotic marker BrdU, we quantify the number of new cells in the glomerular, mitral, and granular layer of the MOB and glomerular, external and internal cellular layers of the AOB 15 days after females were tested in 1 of 4 conditions: (1) females placed in a mating cage (control); (2) females allowed to pace the sexual interaction; (3) females that mated without controlling the rate of the sexual interaction; and (4) females exposed to a sexually active male.

## Results

### Behavioral measures

The student's t-test showed that females in the non-paced group receive more I (t_6_ = −5.401; p<0.01) and E (t_6_ = −2.862; p<0.05) than females that paced the sexual interaction. No statistically significant differences were found in the number of M (t_6_ = 0.899; p = 0.409). There were no differences in M (t_6_ = −0.3594; p = 0.75) or E (t_6_ = 1.640; p = 0.200) latencies. The I latency (t_6_ = −2.678; p<0.05) was higher in the non-paced group. The time between each I that the females received (III) (t_6_ = 3.138; p<0.05) was longer in the paced group. No differences were found in the receptive behavior displayed by the females, as both groups had a similar LQ and MLI. As expected, the percentage of exits was higher after ejaculations than after mounts, and it was also higher after intromissions than after mounts. The mean return latency after mounts showed great variability since only 2 females exited the male compartment during the test. The return latency after ejaculation was higher than that seen after mounts or intromissions (see Table 1).

**Table 1:** Sexual behavior parameters compared (A) in the groups allowed to control the rate of sexual interaction (paced) and in females that had no control over the sexual interaction (non-paced).

A.		
	Groups	
Behavioral measures	Paced	Non-Paced
**Number**		
*Mounts*	2±1	1±0
*Intromissions*	30±2	47±2**
*Ejaculations*	3±0	4±0*
**Latencies (sec)**		
*Mounts*	347.2±145.9	466.2±297
*Intromissions*	769.7±195	1351.5±95.8*
*Ejaculations*	773.1±192.3	439.8±65.5
**III (sec)**	25.3±5	9.4±1.3**
**MLI**	1.90±0.03	1.90±0.05
**LQ**	100	100

B.		
Behavioral measures	Paced	
**Percentage of exits after**		
*Mounts*	12.53±12.2	
*Intromissions*	84.52±7.76	
*Ejaculations*	100	
**Return latencies (sec) after**		
*Mounts*	21.23±14.18	
*Intromissions*	14.68±2.61	
*Ejaculations*	670.78±130.76	

*Different from paced p<0.05;

**p<0.01.

Paced parameters (B). All data are expressed as mean ± SEM.

### Quantitative analysis of new cells

**Figure 1:**
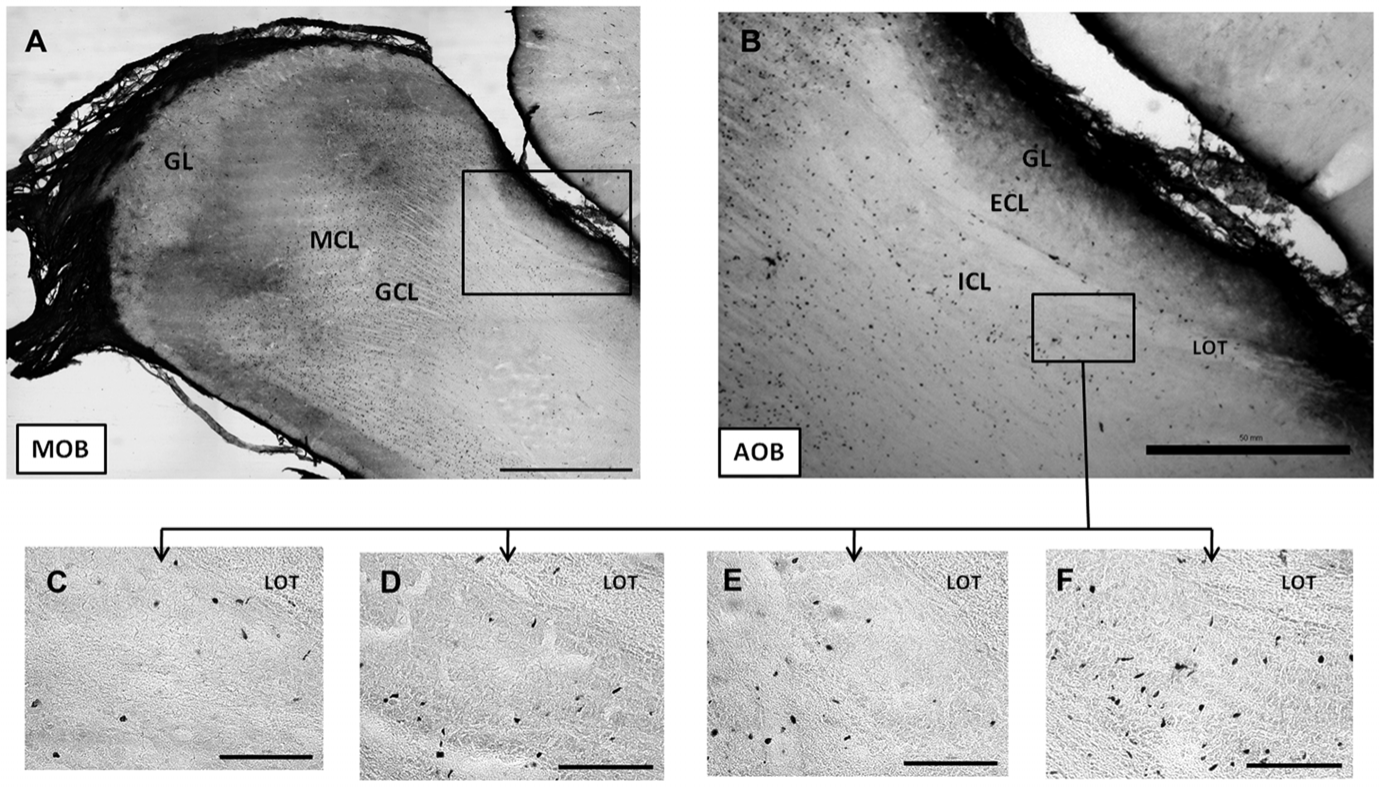
Representative photomicrographs showing BrdU-labeled cells in the A) main olfactory bulb (MOB) and the (B) the accessory olfactory bulb (AOB). A higher magnification of a section in the AOB is shown in the different behavioral conditions (c); C-control, D-exposure to the male, E-non paced and F-paced. See legend of figure 2 for layer description. Scale bars 1 mm (A), 0.5 mm (B), 0.1 mm (C,D,E,F).

**Figure 2:**
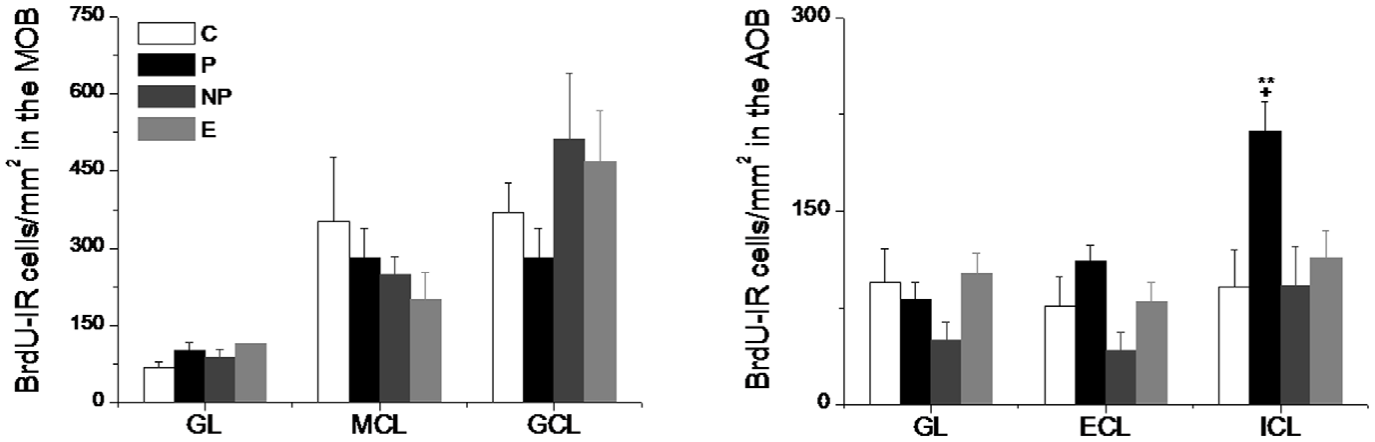
Density of BrdU positive cells in the different groups of animals in the different layers of the main (A) and accessory (B) olfactory bulb. C- control, P- paced groups, NP- non paced and E- exposure to the male. MOB: glomerular (GL), mitral (MCL) and granular (GCL). AOB: glomerular (GL), external (ECL, also known as mitral) and internal (ICL, also known as granular). Values indicate mean density per mm^2^ ± EE from four sections per brain (n = 7 per group). **p<0.01 different from the C and NP groups. +p<0.05 different from E group.

The newly generated cells were quantified as the cellular density immunoreactive to BrdU 15 days after the behavioral test in the glomerular (GL), mitral (MCL) and granular (GCL) layers of the MOB and the glomerular (GL), external (ECL) and internal (ICL) cellular layers of the AOB [34] (see Fig. 1). No differences were found in the GL (F_3_ = 2.7266; p = 0.4357), MCL (F_3_ = 1.8336; p = 0.6077) or GCL (F_3_ = 3.2027; p = 0.3614) layers of the MOB. Furthermore, no differences were found in the GL (F_3_ = 5.8121; p = 0.1211) and ECL layers (F_3_ = 6.8276; p = 0.0776) of the AOB. By contrast, the paced group differed significantly from control and non paced (p<0.01) and from male exposure (p<0.05) in the ICL layer (F_3_ = 10.5507; p = 0.0144) of the AOB, as shown by the post-hoc Mann-Whitney U-Test (see Fig. 2).

**Figure 3:**
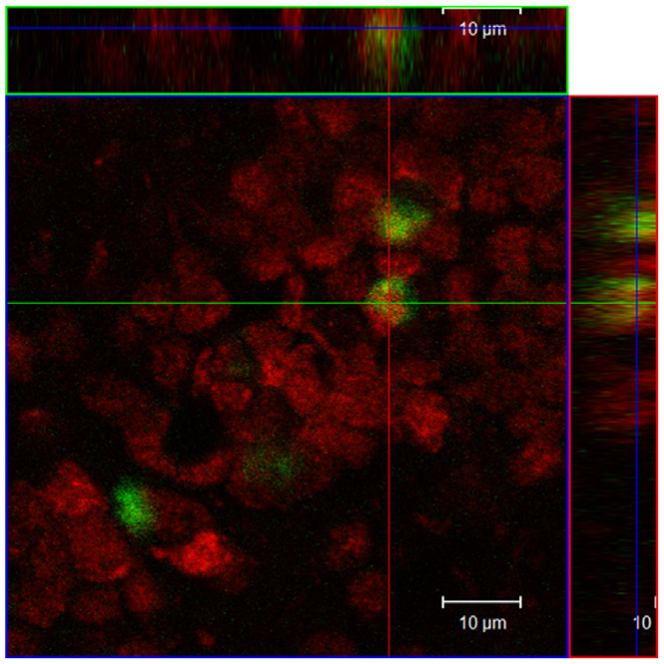
Phenotypic characterization of BrdU-labeled cells in the AOB internal cellular layer (ICL, also known as granular layer) of a female that paced the sexual interaction. The confocal image shows neuronal cell nuclei (red) and new cell nuclei (green) in the ICL. A double labeled cell (BrdU-NeuN) is shown with an arrow in a confocal slice (0.7 mm) in a Z plane with a ortoghonal view demonstrating the presence of a new neuron in the adult female ICL of the AOB.

Since differences in the cellular density of BrdU labeled cells were found in the ICL of the AOB, some sections were processed for double labeled cells in this region. Interestingly we found that a great proportion of the new cells were BrdU-NeuN positive (see Fig. 3).

## Discussion

The results of the present study clearly indicate that pacing behavior promotes a higher cell density of new cells in the internal (granular) cellular layer (ICL) of the AOB. Females of all groups had the same hormonal treatment, and the lordosis behavior of the paced and non-paced groups was similar suggesting that the increase in cell density in the granular layer (ICL) of the AOB is not due to differences in hormone levels or in receptivity. This change is specifically associated with pacing behavior, since non-paced mating and exposure to a sexually active male did not modify the number of cells in the AOB. Interestingly, the females in the non-paced group received a higher number of intromissions and ejaculations than the females that paced the sexual interaction, but this higher frequency of stimulation did not modify the density of new cells. The behavioral differences between the paced and non-paced mating groups that we observed are consistent with results described by others [24], [32], [33]. It is truth that both paced and non-paced mating induce pregnancy and changes that favor reproduction but fewer paced intromissions are required to induce these changes [24] and whenever they have a chance females will pace the sexual interaction in natural, seminatural and under laboratory conditions [24], [28], [32] suggesting that paced mating is qualitatively different. In the light of previous strong evidence indicating that paced mating induces physiological and behavioral changes [24], [25], [27], [28] parsimony will suggest that the paced stimulation that the females receive during mating also enhances the incorporation of new born cells in the AOB.

The fact that changes in the density of new cells were observed only in the AOB is in agreement with several lines of evidence indicating that this brain region is primarily involved in the detection of non-volatile odors like sexually relevant chemosensory cues [35], [36]. Several groups have consistently shown that males and females that mate or are exposed to olfactory relevant chemosensory cues show neuronal activation as evaluated by Fos expression in the olfactory systems [36], [37], [38]. For example, when females of different species mate or are exposed to male bedding or male urine Fos expression increase significantly in the AOB [36], [39], [40], [41], [42]. Also, an increase in Fos expression in the AOB has been described after vaginocervical stimulation [43]. Similar activation of the AOB granule cell layer by Arc (an immediate early gene) was reported after sexual activity and pheromone stimulation [44], [45].

Although it is widely assumed that volatile and pheromonal stimuli activate the main (MOB) and accessory olfatory bulbs (AOB), respectively, more recent evidence obtained in the live hamster, indicates that acute stimulation with either stimuli provoke coordinated activation of both MOB and AOB [46]. Since the pattern of activation is asynchronous (i.e., the MOB is first activated, then the AOB) we may suspect that the repeated pheromonal stimulation in the receptive rat may enhance this differential response in the AOB but not in the MOB [46]. It is suggested that the hormonal environment (i.e., receptivity) and sexual experience [47] coupled with pheromonal stimuli may facilitate or trigger the differential generation of the adult new-born neurons in the AOB observed in the present study.

Other studies have shown that lesions of the AOB reduces lordosis behavior and Fos expression in the medial Me and bed nucleus of the stria terminalis (BNST) after mating in these lesioned females [42], [48]. These results suggest that the AOB and its connections to the Me integrate information to regulate lordosis behavior [48]. Moreover, the Me has been implicated in the vaginocervical neuroendocrine memory formed by VCS that maintains the nocturnal PRL release for two weeks after copulation [26], [49]. This memory is in part regulated by glutamate in the Me, as the pharmacological activation of the Me by the glutamate agonist NMDA initiates a nocturnal release of PRL in females that did not receive VCS [49]. Moreover, PRL itself appears to be increasing cell proliferation in the SVZ and cell survival in the OB [22], [50]. It could be argued, then, that the neurochemical and hormonal changes after the spaced vaginocervial stimulation plus the sexually relevant chemosensory cues received during paced mating could explain the increased density of new cells in the AOB.

Most of the studies of neurogenesis in the OB have focused on the MOB because the majority of odors are processed by this olfactory region, and the largest proportion of new cells born in the SVZ migrate to this brain area. There are several previous studies that clearly demonstrate the relevance of sexual olfactory stimuli on the process of neurogenesis, which include proliferation in the SVZ and in the RMS and the addition of new cells in the MOB. For example, Smith et al. [20] have shown that female prairie voles exposed to a sexually mature male (the females could smell, see, and hear but had limited physical contact with the male) for 48 hours showed behavioral estrus (sexual receptivity), and this behavioral change is correlated with an increase in the proliferation of new cells in the SVZ. In mice, the females that had been exposed daily for 7 days to bedding soiled by male mice showed increased cell proliferation in the SVZ and the granular layer of the MOB [22]. Since we sacrificed the females 15 days after copulation, it is possible that the time frame to observe differences in the number of cells in the SVZ and MOB had passed. After 15–20 days, most of the new cells reach the AOB [17]. In this period the majority of granule cells have already begun to develop spines, dendrodendritic synapses, and are susceptible to cell death or survive in a sensory experience-dependent manner [15], [51]. It has recently been shown that acute and chronic sexual experience increases adult neurogenesis in the hippocampus of male rats that mated once (acute) or for 14 consecutive days (chronic) with receptive females [52]. The female rats that we evaluated had only one behavioral test (exposure to a sexually active male, paced mating or non-paced mating), and we found a higher cell density 15 days after paced mating. To our knowledge there is no other evidence demonstrating that a novel and single sexual behavioral experience promotes an increased density of new cells specifically in the AOB in female rats. Further studies will need to address what happens with the formation of new neurons induced by paced mating in periods longer than 15 days.

To summarize, the present results indicate that a single paced mating encounter increases the number of new cells per mm^2^ in the AOB 15 days after the sexual interaction. Further studies are needed to determine if the neurons survive at different times after the mating experience and the possible factors that could be mediating this response.

## Materials and Methods

### Animals

Twenty eight sexually naïve female rats (*Wistar*) weighing 200–230 g from a local colony were used. They were housed in groups of 3 or 4 rats per cage. All animals were maintained under a reversed 12-h light/dark cycle with food and water *ad libitum*. Female rats were bilaterally ovariectomized under general anesthesia using a mixture of ketamine 70% and xylazine 30% (1 ml/kg per rat). To allow recovery, females were left undisturbed for one week before the experiments. To induce sexual receptivity female rats received a subcutaneous injection of 17-β-estradiol (25 µg/rat– Sigma, St. Louis, MO, USA) and 48 hours later an injection of progesterone (1 mg/rat – Aldrich, St. Louis, MO, USA) both diluted in corn oil. Experimental females were randomly distributed in four experimental groups (*see below*). Sexually experienced male rats (*Wistar*) weighing 300–320 g were used as stimulus in sexual behavior tests. They were trained using other sexually receptive females not used in this experiment. All experiments were carried out in accordance with the “Reglamento de la Ley General de Salud en Materia de Investigación para la Salud” of the Mexican Health Ministry which follows NIH guidelines and were approved by the Bioethics Committee of the Instituto de Neurobiología (INEU/SA/CB/040).

### Behavioral tests

**Figure 4:**
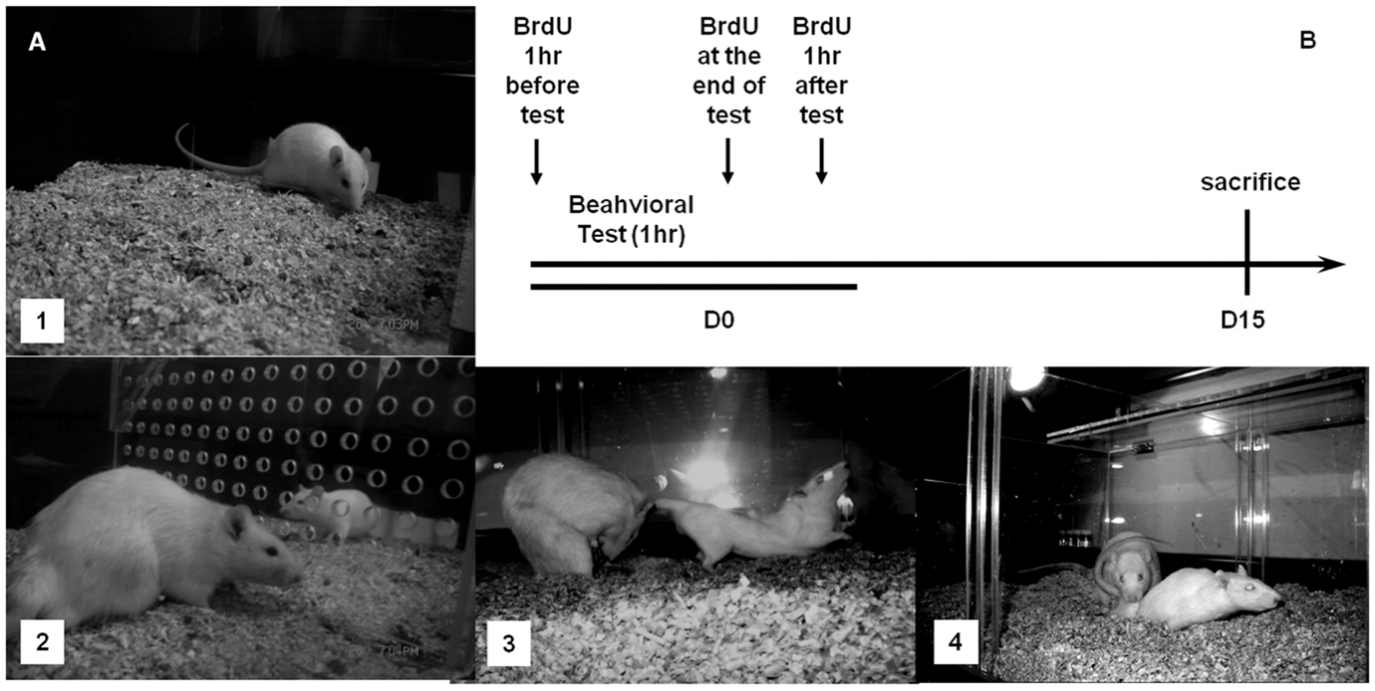
Representation of the different groups (A) and sequence of BrdU injections for all subjects (B). The control group (1) was placed in the mating cage for 1 hr; females in group (2) were exposed to a sexually active male; in group (3) the females mated but could not control the rate of the sexual interaction while females in group (4) were allowed to pace the sexual interaction. BrdU treatment (100 mg/kg per injection; 3 injections) was administered 60 min before the behavioral test (D0), at the end of the test, and 60 min after the test. Subjects were sacrificed 15 days (D15) after the behavioral test.

The behavioral tests were done in Plexiglas chambers (62×29×42 cm). As described above females were injected with estradiol benzoate and progesterone before testing to induce sexual receptivity. The females were divided in the following groups: 1) females placed in a mating cage (control); 2) females allowed to pace the sexual interaction (paced), 3) females that mated but were not allowed to pace the sexual interaction (non-paced) and 4) females exposed to a sexually experienced male (Fig. 4). The control group did not receive any stimuli, but it had the same hormonal conditions and was placed in the testing chamber for 60 min. In group 2, females were allowed to mate with a sexually experienced male in a pacing chamber that allowed the female to control (paced) mating. In this case, the male and female were separated in the test chamber by a removable plexiglas partition with a small area (7 cm in diameter) near the bottom that allows the female to freely move back and forward from the male side. The hole is too small for the male to go through. In the non-paced group the animals mated freely and the male, but not the female, controlled the rate of sexual interaction. Females that were exposed to sexually experienced males were put in test chambers that were equally divided by a removable plexiglas partition with small holes (1 cm in diameter) that allowed the females to smell, see, and hear the male without physical contact. All tests lasted 1 hour. After the test, females were left in their home cages for the next 15 days with other females from the same group (3 or 4 females per cage).

### Behavioral measures

During the sexual behavioral tests (paced and non-paced) different parameters were recorded and calculated: lordosis quotient [LQ; (number of lordosis/(number of mounts + number of intromissions) ×100], and lordosis score (LS), calculated for each lordosis response ranked in an intensity from 0 to 2 according to the extent of dorsiflexion observed, modified from [53]. In order to obtain the mean lordosis intensity (MLI), the sum of lordosis score was divided by the number of mounts plus intromissions received. The number of mounts (M), intromissions (I), and ejaculations (E); the latency to the first mount, intromission, and ejaculation, the postejaculatory interval, and the mean interval between intromissions (III, inter-intromission interval) were also recorded. We also calculated return latencies, defined as the time that the female took to return to the male side after receiving a M or an I, as well as the percentage of exits from the male side after a M or I.

### New cells labeling

The DNA marker BrdU (5′-Bromo-2′-deoxyuridine) (Sigma, St.Louis, USA, dissolved in NaCl 0.9%) was used for the detection of the new cells. It was administered intraperitoneally at a concentration of 100 mg/kg per injection. In order to label all the new cells that were in the synthesis phase of the cellular cycle all the subjects were injected around the behavioral tests. They were injected three times: 1) 60 minutes before the test, 2) immediately after testing, and 3) 60 minutes after the test (Fig. 4). At the end of the experiment, the BrdU positive cells were immunolabeled in the MOB and AOB.

### Phenotypic identification of the new cells

Some alternate sections of the brain were immunolabeled with the NeuN marker, which labels mature neurons [54], plus BrdU marker for the identification of new neurons in the same regions. Most of the sections that contained the main and the accessory olfactory bulb were used to label BrdU cells, unfortunately we didn't have enough sections to make a detailed analysis of double label neurons in all groups.

### Tissue preparation

The animals were sacrificed 15 days after the test and intracardially perfused with 4% paraformaldehyde. The brains were collected with special care to avoid damage to the olfactory bulbs. The brains were placed for 60 min in paraformaldehyde 4% and then transferred to 30% sucrose until histological processing. To detect BrdU-positive cells in the MOB and AOB, floating immunostaining was carried out on 30 µm thick sagittal brain sections obtained with a microtome (Leica). The immunohistochemistry was done on the right and left hemispheres of the brains. The tissue was collected beginning at the MOB and AOB level.

### Immunohistochemestry

The sagittal sections were washed for 20 min in Tris-buffered saline (TBS 0.1 M, pH 7.6) and fixed in 2 N HCL for 60 min. Sections were placed for 15 min in horse serum (20%) containing 0.3% Triton-X (Tx) in a cold room. The tissue was incubated for 16 hrs at 4°C in primary antibody: mouse monoclonal anti-BrdU (1∶2000, BD Biosciences). The sections were then incubated with secondary antibody: biotinylated anti mouse IgG (1∶500, Vector Laboratories, Burlingame CA). The tissue was then incubated in ABC (in 0.32% Tx, Vector Laboratories, Birlingam CA). Bound antibody was visualized with diaminobenzidine (DAB, 0.02%) and 0.01% H_2_O_2_ in 0.1 M phosphate buffer, pH 7.6. The sections were mounted using Permount (Fisher Scientific, USA). Slides were viewed with an Olympus optic microscopy.

Double staining for BrdU and NeuN (neuronal nuclei) were used to identify the neural phenotype of the new cells. Following the protocol described above, the tissue were incubated for 16 hrs at 4°C in two primary antibodies: rat monoclonal anti-BrdU (1∶100, AbD Serotec) and mouse monoclonal anti-NeuN (1∶500, CHEMICON) and revealed using an incubation of the secondary antibodies: anti-rat IgG Alexa Fluor 488 (1∶1000, invitrogen) and anti-mouse IgG Alexa Fluor 568 (1∶1000, invitrogen), respectively.

### Labeled cells and image acquisition

BrdU-DAB stained tissue images (10× objective) were obtained with a light microscope OLYMPUS BX60 and analyzed by Image Pro Plus software. For BrdU-NeuN fluorescence images (multinmersion 25× objective), 0.7 µm confocal slice were scanned sequentially and averaged (30) using an inverted Zeiss LSM 510 Meta confocal microscope (Carl Zeiss).

### Quantitative analysis

**Figure 5:**
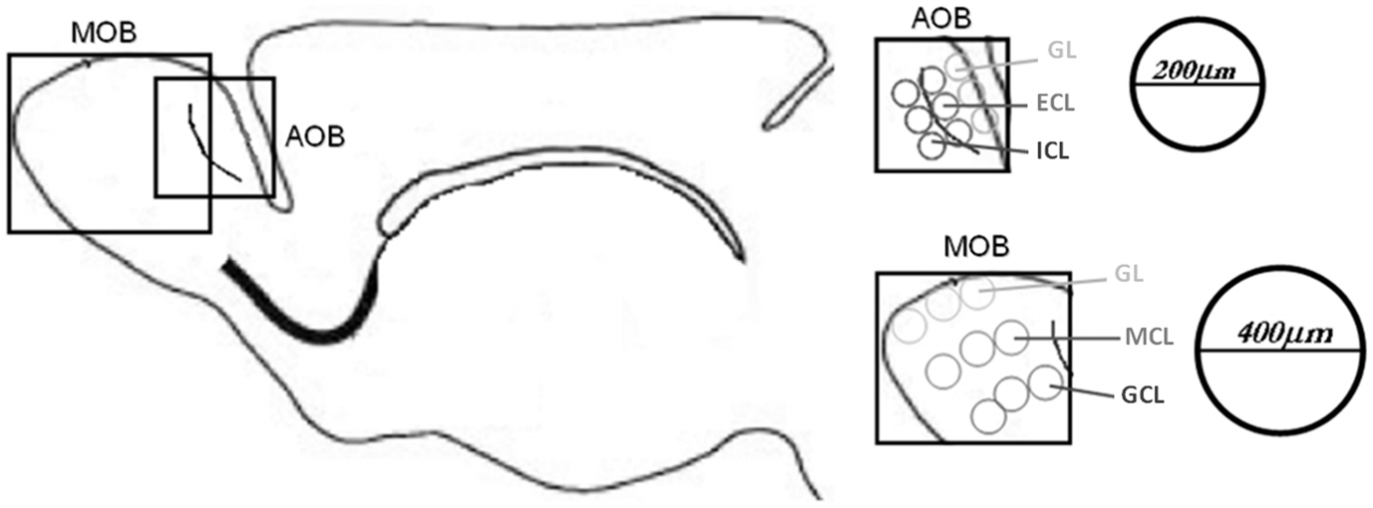
Schematic representation of the regions in which BrdU-labeled cells were quantified in the OB. Left: representation of a sagittal section of the brain indicating the MOB and the AOB. Right: the MOB and AOB open boxes show an amplification of the different layers where the postive BrdU cells were quantified. The diameter of the circles used for the quantification is indicated on the right of the figure. For both structures, the area of interest was delimited with three circles in each layer. AOB: glomerular (GL), external (ECL, also known as mitral) and internal (ICL, also known as granular). MOB: glomerular (GL), mitral (MCL) and granular (GCL) layers.

Positive BrdU cells were identified and quantified in every third section of the glomerular layer (GL), mitral cell layer (MCL), and granular cell layer (GCL) of the MOB and the glomerular (GL), external (ECL, also known as mitral) and internal (ICL, also known as granular) cellular layers of the AOB [34]. Using the Image Pro Plus software 10× images from every region (GL, MCL and GCL) of the MOB and (GL, ECL and ICL) AOB were taken for quantification. For both structures the area of interest was delimited with three circles in every layer (Fig. 5). The diameter of the circles used in the MOB was 400 µm, and for the AOB it was 200 µm. The total area of interest per layer was calculated with the sum of areas in each circle. Cellular density was obtained with the average number of BrdU positive cells in the total area of interest per layer. We quantified four sections per brain (n = 7 subjects per group).

### Statistical analysis

We used a Student's t-test to compare the different behavioral measures, number and latencies of M, I, and E, as well as the III between the paced vs the non-paced group. These behavioral measures were normally distributed. The statistical analysis for the quantification of density of the BrdU-labeled cells was done by a one way-ANOVA Kruskal-Wallis test followed by a Mann-Whitney U-Test. One ANOVA was done for each layer of the main and accessory OB. A non-parametric test was used, because there was no homogeneity of variance in the cellular density among the different groups.
